# Voluntary medical male circumcision for HIV prevention among adolescents in Kenya: Unintended consequences of pursuing service-delivery targets

**DOI:** 10.1371/journal.pone.0224548

**Published:** 2019-11-04

**Authors:** Adam Gilbertson, Barrack Ongili, Frederick S. Odongo, Denise D. Hallfors, Stuart Rennie, Daniel Kwaro, Winnie K. Luseno

**Affiliations:** 1 Pacific Institute for Research and Evaluation (PIRE), Chapel Hill, North Carolina, United States of America; 2 UNC Center for Bioethics, University of North Carolina at Chapel Hill, Chapel Hill, North Carolina, United States of America; 3 Department of Social Medicine, University of North Carolina at Chapel Hill, Chapel Hill, North Carolina, United States of America; 4 Kenya Medical Research Institute (KEMRI), Kisumu, Kenya; University of Washington, UNITED STATES

## Abstract

**Introduction:**

Voluntary medical male circumcision (VMMC) provides significant reductions in the risk of female-to-male HIV transmission. Since 2007, VMMC has been a key component of the United States President’s Emergency Plan for AIDS Relief’s (PEPFAR) strategy to mitigate the HIV epidemic in countries with high HIV prevalence and low circumcision rates. To ensure intended effects, PEPFAR sets ambitious annual circumcision targets and provides funding to implementation partners to deliver local VMMC services. In Kenya to date, 1.9 million males have been circumcised; in 2017, 60% of circumcisions were among 10-14-year-olds. We conducted a qualitative field study to learn more about VMMC program implementation in Kenya.

**Methods and results:**

The study setting was a region in Kenya with high HIV prevalence and low male circumcision rates. From March 2017 through April 2018, we carried out in-depth interviews with 29 VMMC stakeholders, including “mobilizers”, HIV counselors, clinical providers, schoolteachers, and policy professionals. Additionally, we undertook observation sessions at 14 VMMC clinics while services were provided and observed mobilization activities at 13 community venues including, two schools, four public marketplaces, two fishing villages, and five inland villages. Analysis of interview transcripts and observation field notes revealed multiple unintended consequences linked to the pursuit of targets. Ebbs and flows in the availability of school-age youths together with the drive to meet targets may result in increased burdens on clinics, long waits for care, potentially misleading mobilization practices, and deviations from the standard of care.

**Conclusion:**

Our findings indicate shortcomings in the quality of procedures in VMMC programs in a low-resource setting, and more importantly, that the pursuit of ambitious public health targets may lead to compromised service delivery and protocol adherence. There is a need to develop improved or alternative systems to balance the goal of increasing service uptake with the responsible conduct of VMMC.

## Introduction

Voluntary medical male circumcision (VMMC) provides significant reductions in the risk of female to male HIV transmission.[1–3] Since 2007, VMMC has been a key component of the Joint United Nations Program on HIV/AIDS (UNAIDS) strategy for ending AIDS by 2030 and a top priority of the United States Agency for International Development (USAID) and the United States President’s Emergency Plan for AIDS Relief (PEPFAR) to combat HIV in sub-Saharan Africa.[4, 5] Between 2007 and 2018, almost 19 million PEPFAR-supported circumcisions were performed in 14 priority World Health Organization (WHO) designated countries in eastern and southern Africa (ESA); 10% (more than 1.9 million) of these MCs took place in Kenya.[6] From early on, Kenya was ahead of other countries in VMMC scale-up, meeting 34% of its target in 2012 compared to 16% achieved by the next best country.[4] With a consistent record of meeting national VMMC uptake goals that sets it apart from other countries,[7] Kenya is often presented as a leading VMMC success story in ESA.[8]

The United States (US), via PEPFAR, took an early lead in funding VMMC for HIV prevention programs in ESA. In Fiscal Year (FY) 2016, PEPFAR spent over 15 million US dollars (USD) for VMMC services in Kenya alone.[9] These funds are distributed to VMMC implementing partners (IPs) in Kenya and other ESA countries to establish and maintain VMMC services within designated catchments in regions of high HIV prevalence and low levels of male circumcision (MC).[4, 10, 11] These IPs include non-governmental organizations (NGOs), community-based organizations, faith-based organizations, or other non-profit or for profit entities. Each IP is accountable to its funding body via in-country intermediates and is required to keep accurate records and to make regular reports concerning the services provided, numbers of circumcisions performed, and observed adverse events.[12]

As a part of this process, epidemiological model-informed quotas or “targets” are set by funders for the number of MCs to be performed within a given timeframe (e.g., annually). In FY 2016, 264,490 PEPFAR-funded MCs were performed in Kenya,[13] thus exceeding the FY 2016 target of 240,000 MCs.[14] In Kenya, PEPFAR has identified 11 VMMC underserved, priority counties.[14] In FY 2017, the coverage rates for 15–29 year-old males among all priority counties were reported to be approaching or to have reached 80%,[14] though more recent studies have called into question these coverage estimates.[15] To achieve this goal of 80% MC coverage within all these underserved areas by 2019, PEPFAR Kenya set a target of 300,000 new MCs in FY 2017, a 25% increase from the FY 2016 target.[14] In 2014, Kenya began offering VMMC services to boys aged 10–14 years citing “high demand for MC amongst this age group”, cultural preference, and pre-sexual debut and/or less sexual activity as the rationale.[16] Between October 2016 and September 2017, 227,272 PEPFAR-sponsored MCs were performed in Kenya; 60% of these were among boys aged between 10–14 years, 23% were among 15–19 year-olds, and 14% were among 20–29 year-olds.[13] Among all priority-designated counties in Kenya, UNAIDS estimates cited by PEPFAR suggest that an additional 290,000 MCs may be needed to achieve 80% MC coverage among 10–29 year-olds, or an additional 500,000 to achieve 90% coverage by 2021.[14]

Previous research has highlighted ethical and practical concerns associated with VMMC implementation for HIV prevention.[17] Among school-going adolescent and young adult males in ESA, these concerns include recruitment practices (referred to as mobilization in VMMC programs),[18] consent and assent,[18–20] and access to follow-up care.[21] Additional issues include adolescent understanding of VMMC’s purpose,[22] MC’s relevant benefits and risks,[19, 20, 23] and the importance of HIV protective behaviors following circumcision.[24] A 2014 assessment of VMMC quality of care across four countries (Kenya, South Africa, Tanzania, Zimbabwe) identified deficiencies including providers’ failures to adhere to national VMMC best-practice guidelines and a lack of equipment and supplies at VMMC clinics.[25] Similarly, a 2017 report prepared for review by USAID identified multiple “quality gaps” in VMMC implementation, including adverse events, low client follow-up rates, and inconsistent messaging to clients.[26]

During research for a parent study on the responsible conduct of HIV research in adolescent populations in Kenya we received anecdotal reports from local health leaders and community stakeholders that raised concerns about the ethical implementation of VMMC among adolescents. Given the known ethical and practical concerns linked to VMMC implementation across ESA, we set out to conduct a qualitative study to learn more about VMMC practices “on the ground” in Kenya. Presented below, an analysis of the qualitative data we collected revealed that the pressure to meet funder-set service-delivery targets appears to generate some unintended consequences concerning the services provided and/or standards maintained by VMMC IPs.

## Methods

HIV prevalence in Kenya in 2017 was 4.8% overall among individuals aged 15–49, with a range at the county level of between 0.1% and 21.0%.[27] This study was conducted in an area of Kenya with elevated rates of HIV and historically low uptake of MC. From March 2017 through April 2018, we conducted in-depth interviews with 29 VMMC stakeholders (21 males and 8 females), including schoolteachers, program recruiters (known locally as “mobilizers”), HIV counsellors, clinical providers, and policy professionals (e.g., local VMMC technical advisors). Table 1 lists these stakeholder interviewee categories, the number of stakeholders from each IP or school, and descriptions of each category’s role or links to VMMC program implementation. In addition to these interviews, we conducted field observation sessions at VMMC clinics located in the research area.

**Table 1:** Stakeholder interviewee categories, number of interviewees and their employers, and descriptions of stakeholders’ involvement in VMMC.

Stakeholders	Interviewees/ employers	Involvement in VMMC
**School teachers** **N = 6**	5 primary schools and 1 high school	Many adolescents are mobilized for VMMC at school; teachers facilitate access to students for VMMC mobilizers, are a source of information for students and parents, and often help to distribute and collect consent forms.
**Mobilizers** **N = 9**	IP A: 5 IP B: 3 IP C: 1	Mobilizers are recruiters of VMMC “clients” (individuals who receive VMMC services). They are paid to conduct informational outreach activities, including “health talks” in schools, and to ensure that VMMC facilities have access to adequate numbers of clients to meet targets.
**Counselors** **N = 4**	IP A: 2 IP B: 2	VMMC counsellors inform boys about VMMC benefits and risks, as well as post-circumcision wound care and healing best practices. They oversee consent and assent and conduct HIV counselling and testing.
**Clinical providers** **N = 7**	IP A: 4 IP B: 1 IP D: 1 IP E: 1	Clinical providers perform circumcisions and provide post-procedure care at VMMC facilities. They include clinical officers (surgeons), nurses, and infection prevention officers. Nurses assist clinical officers during circumcisions. Infection prevention officers oversee the surgical instruments, sanitary practices, and waste collection and disposal.
**Policy makers N = 3**	IP A: 1 IP B: 1 GOK: 1	Policymakers are individuals involved in, or with in-depth knowledge concerning, VMMC practices and policies in Kenya.

VMMC, Voluntary Medical Male Circumcision; IP, Implementation partner; GOK, Government of Kenya.

To recruit stakeholders for interviews, our Community Liaison Officer asked for assistance from authorities at the regional Ministry of Health, Ministry of Education, and Health Department. Additionally, we sought approval from six VMMC IP NGOs to conduct interviews with their staff members and observation sessions at their clinics (see Table 1). Two of these IPs approved our request to conduct interviews and observation sessions (IPs A and B). Three of these IPs gave their approval for interviews but declined our request to conduct observation sessions (IPs C, D, E). One IP declined to approve both interviews and observation sessions. These authorities and IPs assisted us to identify local VMMC professionals with at least three years of experience related to policy, clinical services, counselling, or mobilization. In turn, VMMC mobilizers helped us to identify teachers involved with in-school VMMC mobilization activities referred to as “health talks”. Once identified, our Community Liaison Officer and study coordinator (FSO) contacted potential participants in person or via telephone and inquired regarding their willingness to take part in an interview. Out of the 38 stakeholders contacted, 32 agreed to take part in an interview; of these, 29 were available to attend the interview. Out of the 23 mobilizers, counsellors, clinical providers, and policy professionals interviewed, 12 were employed by IP A, seven by IP B, one by IP C, one by IP D, and one by IP E (see Table 1). An additional policy professional interviewee was employed by the Government of Kenya. The six teachers we interviewed came from five different local primary schools and one high school.

Based on preliminary fieldwork in the region in 2016, we developed interview guides for each stakeholder category via an iterative process that relied heavily on the input and experience of our Kenyan team members (WKL, BO, FSO, DK). These guides included questions related to careers, roles, and experiences with VMMC, use of targets/quotas, clinical/mobilization protocols and practices, record keeping/documentation, informed consent, HIV testing, benefits and risks, training, the structure/hierarchy of VMMC IP organizations, and remuneration (see S1 File).

Study participation was voluntary and all participants gave consent before interviews began. Stakeholder interviews were conducted by AG in English (Swahili and English in one interview). These interviews took place in quiet, secluded locations, lasted up to 90 minutes, and were audio-recorded with permission. Kenyan team members who are fluent in English, Swahili, and the local language listened to the audio-recorded interviews and translated and transcribed them in English. The transcription process involved three steps. First, audio-recorded interviews were transcribed verbatim. Second, the transcriber conducted quality assurance by reading the transcript while re-listening to the audio. Third, the transcriber submitted the English transcript and the audio file to the Study Coordinator (FSO) for final review and designation as the final version of the transcript. Any portions of interviews that contained Swahili were first transcribed in Swahili before being checked, translated to English, and rechecked for accuracy by the Study Coordinator. Each interviewee received 500 Kenyan shillings (approximately 5 USD) for their time and transportation.

A Kenyan nurse and public health worker who is knowledgeable about VMMC clinical procedures and fluent in English, Swahili, and the local language (BO) was our primary field observer. He conducted two- to five-hour observation sessions at 14 VMMC clinics run by the two NGOs (six administered by IP A and eight by IP B) and alongside the mobilization team activities at 13 community venues (six from IP A and seven from IP B). These venues/activities included two schools (health talks), four public marketplaces (mobilization among local motorcycle taxi drivers), two fishing villages (informational speeches, music, skits; referred to as “roadshows”), and five inland villages (door-to-door adolescent mobilization). The nurse/public health worker documented what he observed in detailed field notes. A medical anthropologist (AG) supervised observation activities.

We identified four health facility levels (levels 2, 3, 4, and 5) for VMMC observation. Level 2 facilities include dispensaries and small clinics usually run by nurses that provide basic outpatient care to up 10,000 people. Level 3 facilities are health centers that serve populations up to 30,000 and are staffed by midwives, nurses, clinical officers, and occasionally doctors. They provide basic curative and preventive, minor surgical, and reproductive health services. Level 4 facilities are primary, high-volume referral hospitals that serve populations of 100,000 people. Services offered include curative and preventive care and surgeries and emergency services that are not available at smaller facilities. Level 5 facilities serve larger populations. They focus on specialized services and provide clinical supervision and support to lower level, primary referral facilities.[28, 29]

Our Kenya-based research team selected VMMC clinics to ensure that we conducted observations at a variety of facility levels in various sub-counties, as well as during relatively active (more than 30; up to 100 or more) and slow periods (less than 15) circumcisions per day. Out of the 14 VMMC facilities we observed, two were Level 2, six were Level 3, five were Level 4, and one was Level 5. Upon arrival at each facility, BO, our Community Liaison Officer, or our Study Coordinator (FSO) informed facility staff members about our research and provided them with details concerning their IP’s approval for our observations to take place before they gave verbal consent. Observation sessions tended to coincide with the arrival of adolescent VMMC clients and their subsequent intake, counselling, testing, and circumcision. The mobilization teams associated with these clinics were identified and approached outside of the clinical setting for permission to observe their activities (e.g., mobilization events at schools or public markets). Before these observation sessions began, members of the mobilization teams gave their verbal consent to be observed. Prior to beginning in-school observation sessions, school authorities and teachers were informed about the research and gave us permission to conduct the research activities.

The institutional review boards of the Pacific Institute for Research and Evaluation (PIRE) and the Kenya Medical Research Institute (KEMRI) approved all study activities, including all the informed consent procedures described above. Locally, the county-level Ministries of Health and Education and the directors of each IP gave us approval to conduct this research.

### Analysis

Kenyan team members translated and transcribed the audio-recorded interviews and checked the final transcripts for accuracy. U.S.-based team members conducted an early analysis of interviewee transcripts and observation-obtained field notes. The potential for the pursuit of VMMC targets to contribute to unintended consequences emerged during early data analysis. We created the codebook to define and identify themes related to the use of targets in VMMC policy and service delivery.[30] Two team members then independently reviewed and coded the full set of transcripts and field notes using MAXQDA 12.[31] Any coding discrepancies were discussed and resolved by the two coders. Code reports were produced and reviewed, and quotes were selected to illustrate typical responses for each theme. We present these quotes below, along with corroborating field observations, and participant demographics and identification numbers.

## Results

To meet VMMC quotas, local IPs communicate target numbers to their VMMC clinics and to the mobilizers who are responsible for supplying these clinics with clients to circumcise. The numbers of adolescent clients who undergo VMMC vary across the year, depending on the relative availability of clients and/or the ability of mobilizers to access them.

### Implications of the pursuit of VMMC targets

Data collected from the stakeholder interviews and field notes suggest that funder-designated targets for VMMC service delivery effectively motivate mobilizers and clinic staff. As one 33-year-old, male mobilization supervisor put it, “…target or quotas act as the engine to make us do a lot of work and ensure that there is continuous [flow of clients]” [Mobilizer 1]. For mobilizers especially, monthly remuneration (and their future employment) depends on referring enough clients to the clinic each week; failing means earning less, regardless of the amount of time and effort invested in mobilization. From our interviews we learned that while most mobilization supervisors receive a base salary from their IPs regardless of the targets, other lower-level, subcontracted “associate” mobilizers or “peer educators” only receive payment by providing clients and meeting targets. Describing the competition this system fosters among mobilizers, a 28-year-old, male community-level mobilizer explained, “…we are paid upon reaching the target […] so we are really competing, until somebody may grab your clients before you know it” [Mobilizer 2]. For VMMC clinics, achieving targets is essential to the continued funding of their respective IPs. A 49-year-old, male mobilization supervisor offered, “…targets… are about funding, and [if] you don’t reach your target, you don’t get funding” [Mobilizer 3]. However, data analysis revealed that beyond motivating staff members, the pursuit of targets may introduce problems for VMMC implementation. Presented below, these areas of concern relate to the ebb and flow periods of VMMC client availability (referred to with reference to agricultural “seasonality” elsewhere),[32, 33] and the resulting increased burdens on clinic resources and staff, long waits for clients, potentially misleading or questionable mobilization practices (including possibly undue inducements), problematic uses of social pressure, and circumcision of children under age ten. By undue inducements, we mean benefits (monetary or non-monetary) that are used to motivate prospective participants (in this case adolescents or young adults) to join a study—benefits that may be inappropriate because of their potential to distort a participant’s understanding of the study.[34] A final issue that we link to the pursuit of targets is the reduced quality of clinical care.

### Variations in VMMC client availability: “High and low seasons”

Adolescent client availability for circumcision rises and falls throughout the year, primarily due to academic schedules: schools do not allow VMMC mobilization to take place on school grounds near the time of exams or other especially busy periods in the year. This results in “high” and “low” seasons for VMMC among these age groups. As Mobilizer 1 explained:

There are times when they are having exams, or when exams are approaching, or when there is athletics or ball [soccer]; when those are taking place, we don’t get people coming… we end up having zero clients cut [circumcised]. And you know, when there are zero clients cut, all the pressure comes to the mobilization supervisor because the surgeons will be saying, ‘We are ready to cut, where are the clients?’ So the buck stops on me as the mobilizer; so it is not a very easy task.

Likewise, a 30-year-old, female mobilization supervisor offered, “We bank on schools so much. And now, when they are taking their exams, you can’t pick a child from school [….] …when schools are so engaged, then definitely I will not meet that target” [Mobilizer 4]. Recognizing the need to strategize VMMC service provision around local school schedules (and client availability), some IPs may institute “Rapid Results Initiatives” or similar short-term drives aimed at increasing dramatically the number of MCs conducted during school holidays.[8, 35, 36] If funding is insufficient, or if IPs are otherwise unable to commit the resources (e.g., personnel, transportation, clinics, and medical supplies) needed for these drives, then targets may go unmet.

### Increased burden on VMMC facilities and staff

**Fig 1:**
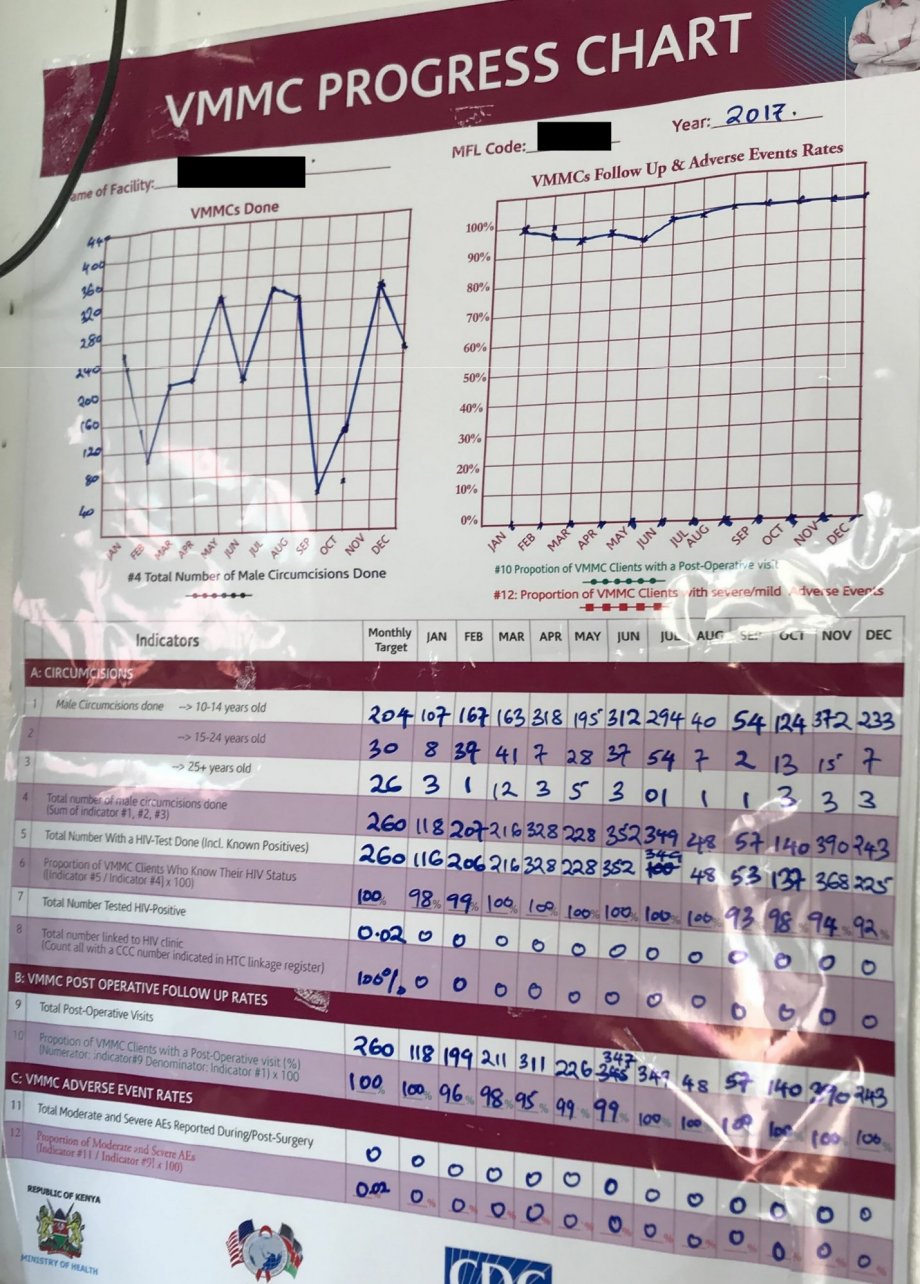
VMMC Progress Chart for 2017 displayed on the wall of a clinic.

VMMC clinical teams are generally comprised of at least one surgeon, surgical assistant, counsellor, and infection prevention officer.[16] During the high season, efforts to meet targets may push some teams to circumcise more clients than the approved quota to be conducted per day per team. During the 2009 Rapid Results Initiative, the projected average number of circumcisions per team per day was set at 12, and the achieved average was 10.2 (with a range of 8–12).[36] In 2018, we observed an IP internal document dated December 2017 that was posted to the wall in a public space of one of the clinics in which we conducted observations. This memo made clear that the IP was aware that “some teams are circumcising over and above the safe maximum number of clients per team” and that this limit is 25 clients per day. During one observation session on a high season day at a Level 4 facility, we counted over 100 boys waiting to be circumcised at a clinic with four surgical beds. At this same facility in the low season, less than half this number may be circumcised in a month (see Fig 1). On days when an unexpectedly high number of boys are mobilized for circumcision, VMMC teams may be left scrambling to call in additional staff. As one 39-year-old, male Clinical Officer explained: “If the number is high, you have to get those who are locum [temporary, fill-in staff]; for instance, like today, if the number is beyond 60 you have to get those for locum because one counselor cannot handle 60 clients. So we do arrangements prior and we talk to the office and ask them to give us assistance [Provider 1]”

Referred to as “moonlighting” by our interviewees, VMMC staff may sometimes work extra hours, late into the evening, and/or over weekends or holidays when they would not normally be expected to work (for which they receive extra compensation) in order to handle large client loads, meet current targets, or make up for previous months when a lack of clients meant targets were missed. One 33-year-old, female counselor explained, “Yeah, you are put into pressure and you have to get the targets. We always know we have to get our targets, come what may. So we do moonlight to get those targets” [Counselor 1]. Similarly, a 39-year-old male Clinical Officer told us: “…if the number is high sometimes we can extend to weekends; and based on the clients flow like now… schools are going to be closed, and hence the number of clients is going to be high. So tomorrow [Good Friday] we may work” [Provider 2].

While severe adverse events (AEs) beyond minor swelling, bleeding, or infection are rare,[37] when they do occur a few of our interviewees suggested that provider fatigue may be a contributing factor.[38] Referencing one case, a 42-year-old male VMMC Clinical Officer offered:

Then the other factor is the high numbers given as targets annually. So if you look at the number of clients you should do, you get fatigued along the line because remember you stand all day. So if you do 20 clients or 25 clients in a day, every day, say from Monday to Friday, it is a hell of a tiresome job…. So most of those AEs [are] attributed to fatigue, because if you look at the timings of most of the AEs, it is usually late in the evening, after lunch, afternoon; so it has always been attributed to high volume in terms of target, and then fatigue, then people not following the protocol. You find somebody, because he wants to finish faster, they want to split [meaning the surgeon and the assistant each operate on their own] instead of [following] MC protocol [and working together]…. It reaches a point where you have to split where everyone is doing [operating alone] because the number is high…. [Provider 3]

### Long waits at VMMC clinics

Another unintended consequence of targets for school-aged clients is the long waits they may experience at VMMC clinics before and after MC, especially during the high season. Despite mobilizer claims about the short duration of the procedure, inadequate numbers of vehicles to transport boys, rough road conditions (especially during the rainy season), and sometimes understaffed and under-resourced VMMC clinics mean that adolescents may spend hours waiting to be circumcised and then to be ferried back home. While making observations at one Level 3 facility, boys arrived for circumcision at approximately 8am; due to the staff’s late arrival (and possibly understaffing), circumcisions began at 12.30pm. At 4.15pm, when we left, these children were still waiting at the facility for transportation home. Some of the children were crying and saying that they want the pain “to find them at home”. One child stated, “We were told it will only take 20 minutes. This is not 20 minutes!” According to one 32-year-old, male primary school teacher, “…one day I attended a facility and [found] that a number of people are to be cut, but the officers who are doing this are just two. So they will do it up to very late [in the evening] and these boys are just there hungry, just with one bottle of soda which will not sustain them” [Teacher 1].

### Suggestions of misleading or questionable mobilization practices

Data collected during interviews and observations suggest that the drive to meet targets may lead some VMMC mobilizers to use misleading or otherwise questionable mobilization practices to increase the number of adolescents they can refer for circumcision. As Mobilizer 1 explained:

Another thing is the peer mobilizers, yeah, someone wants to meet his or her targets and that is the time that they engage in what I called the uncouth methods, whereby the end justifies the means; whichever means that can make those clients come out. So you end up having the clients coming, but they are not coming for the VMMC the way we want it. They have been pushed; they have been coerced…yeah. This one is happening because of these targets. So that is the downside of the targets.

These practices are sometimes blatant: during one door-to-door village mobilization, the mobilizer kept referring to himself as “doctor” although he had no medical training. Another mobilizer, a 38-year-old male who referred to himself as a “VMMC champion” explained how he pays boys to help him mobilize their friends:

For us we are calling it ‘broking [brokering] system’. So after the exercise, you tell him to go… and convince a friend and bring a friend… [….] For me I am using my own system being that we are not even allowed to give children money. But for me, because I have a target, and if I get my target, I am expecting 10800 KES [108 USD]. So I do divide this 10800 KES; maybe if he brings me two boys, I can give him up to 50 KES. I can even give out 100 KES [1 USD]. The moment I give that money, 50 bob, to that boy, he is going to bring me more four boys. So the more he bring boys for instance if they are four, I give 100–150 KES. At the end of the day, I will hit my target [Mobilizer 5].

Other times, these practices are subtle and easy to overlook. During a promotional skit in which mobilizers played the roles of a husband, wife, and brother all discussing VMMC, the message to the audience was one of strong female preference for circumcised men. The wife told her husband, “Go and cut your *firimbi* [whistle] … go and remove that sleeve of a sweater. That whistle is not going inside me…don’t blow the whistle inside me. I have refused…. I am not giving you [sex].” Later, when the husband’s brother arrived, he explained that his wife is refusing to have sex. In response, his brother said, “I was passing here to tell you to go for circumcision. Look at me, I have gone, and I am fine. I am now a clean person.” Explaining the benefits, his brother offered that MC reduces HIV risk by 60% and enhances cleanliness (“you avoid smelling”) and prevents cervical cancer.

While VMMC reduces the risk of HIV acquisition by 60%[39–41] or more,[42, 43] it may be confusing to suggest that MC reduces an individual’s risk of infection by 60% if the differences between absolute and relative risk are left unexplained (as we observed). Similarly, while studies suggest that female preference for circumcised males exists,[44–46] it may also be misleading to generalize about female preferences during recruitment efforts since the individual partners of the boys and men in the audience may think differently. Furthermore, though MC does reduce the risk of human papillomavirus transmission from men to women, thereby reducing their risk for cervical cancer,[47] this messaging may be unbalanced or misleading if the audience comes away thinking that women whose husbands are circumcised do not or cannot get cervical cancer.

This practice of emphasizing the benefits (including reducing the time needed to bathe and risk of acquiring penile cancer) and incentives related to MC while downplaying the risks was common among our participants. The most often discussed risks included temporary pain, minor swelling, and bleeding, while the Government of Kenya VMMC consent form (see S2 File) in use at this time lists risks including bleeding, swelling, pain, infection, injury, numbness, sensitivity loss, mutilation, amputation, and HIV infection. These latter, more severe but rarer adverse events did not feature in the health talks and other mobilization activities we observed.

According to interviewees, food, money, water, sanitation, clothing, and other basic needs are issues of concern for many adolescents’ households. During health talks, some mobilizers emphasized the incentives that boys will receive (e.g., sodas and/or new underwear) following circumcision—items that may be significant inducements among impoverished children. During a village mobilization effort, one mother explained her reluctance to let her son be circumcised: “…this one is small. Even his heart was there because those who have come from circumcision are ‘seducing’ their friends to go also. They are telling them, ‘go and it is not painful.’ They are also giving people sodas. So that soda is what brings them nearer. I told this boy not to sneak, but to wait and I will take him there….” Offering a similar analysis, Provider 3 told us:

Sometimes they also entice them for us; in the facility we give them a bottle of soda, Fanta to be specific because one, some of the boys come probably from poor families, they haven’t had a meal in the morning or breakfast and sometimes stay in the clinic for more than two hours, three hours and thus to maintain their sugars. [But] that one [soda] becomes a bait, especially for the young ones ‘We will give you a soda; we pick you with a vehicle’ …which is actually wrong because, it is like enticing a client which you are not supposed to, but that is how they [mobilizers] ply their trade.

However, according to our interviewees and as we observed first hand, clinics sometimes lack sodas and/or underwear to give to clients.

### Social pressure

All six of the teachers we interviewed recognized social pressure as a primary motivator for MC among adolescents. During observations of mobilization activities, we saw firsthand how mobilizers sometimes make use of or encourage peer pressure to promote circumcision, including through the use of abusive and/or stigmatizing language. During a health talk in front of a large group of boys at a primary school, one male mobilizer referred to foreskins as the “sleeve of a sweater” and “cold *matumbo”* [boiled goat intestines]; at the end of the talk, he implored those who still had their “whistles” to have them removed. He also told them: “Those who are still having the foreskin, you are the ones who are going to spread the diseases. In the future, you are the ones who are going to spread the HIV virus.” Later that same day, the mobilizer asked a large group of older students,“How many of you wish to get the HIV virus? If you don’t want to get the HIV virus, the only option is to remove the foreskin.” Revealing his awareness of this type of language and messaging within VMMC mobilization, Provider 3 explained:

This is demeaning and they try to create some peer pressure. Like when you go to those mass cuts in high schools, probably they registered like 100 clients, you would likely get another probably 30 or so who, because of the peer pressure, just jump into it. But if it was like walking from home to hospital and demanding for the service, they are not likely to have gone. But now that it is here, and so and so has gone and is my friend, I have to be in it.

### Circumcision of children

Current VMMC for HIV prevention guidelines and national protocols approved for use in Kenya require that adolescent clients must be at least ten years of age.[16] Despite this directive, interviews and observations confirmed that VMMC staff sometimes mobilize and circumcise boys who are under ten years of age in order to boost their numbers to meet targets. When asked about potential links between targets, mobilizer strategies, and underage circumcision, Provider 3 offered:

Yes, they bend the rules… because you know sometimes our current age according to WHO is 10 years and above. But when they [mobilizers] go to the field and get a nine-year-old for example, they coach this client and the parent, because we insist on seeing the consent. We always insist they put the ID and telephone number of the parent [on the consent form]. When they come to the facility, we [call to] confirm the age and whether the parent has consented for the child to be circumcised. And you know, they will tell you ‘yes I am 10 years old’, but when you look at the guy, he is probably 8 or. . . .

We observed evidence for the mobilization of underage boys at ten of the 14 VMMC facilities we visited for observation, including boys (and their mobilizers) who readily admitted that they were underage. However, as Mobilizer 1 noted, age verification is not easy or straightforward in Kenya: “Yes, we have had some cases where children try to adjust their age. For instance, he is eight years old, but insists he is ten years old; and they are even able to calculate that they were born on this date and in this year. Being that we don’t have a way to verify that… then later you realize that this boy is underage.” Yet, during observations made during school health talks, as well as during community-based door-to-door mobilization activities, we frequently observed mobilizers not asking boys their ages, opting instead to ask for their class in school as a proxy for their age.

### Reduced quality of clinical care

Under pressure to meet targets, some Clinical Officers and other VMMC medical staff find ways to speed up the clinical process. For example, as we were told during interviews and observed firsthand at multiple VMMC facilities, providers may opt not to take patient medical histories or conduct preoperative examinations (e.g., blood pressure, weight, preexisting conditions, etc.) and/or 30-minute post-operative check-ins, and yet fill in the requisite forms as if these activities had been completed. Other avenues for time-saving include rushing circumcisions, inadequate stitching, splitting surgical utensil and bandage packs between patients, not following recommended protocols or procedures (e.g., the dorsal slit method for circumcision), and stacking patients one after another with little pause in between, which raises additional sanitary and privacy concerns. According to an experienced VMMC surgeon:

We cut a lot of corners and I will give you an example: […] so because of speed, you find that while one client is dressing [after circumcision], the other one is [already] undressing. Ideally, it is supposed [to be] that you finish with this client, you give [them] instructions on how to take medication and all that, but because of the high volume, you find that some of us we are not the ones giving out the medication. We assign somebody randomly, you know, who will be giving out the medication and the refreshment and so [the] information [they provide to the client] may not be correct. [….] Sometimes even the vitals of post-operation 30 minutes after [circumcision] are not done. It is skipped, or somebody else does [it]—probably a receptionist who is nonmedical, just to fulfill the requirements of the form. [This] is wrong because you are supposed to do the circumcision and the client rests for 30 minutes in a bed or a couch, then after that, you do the vitals then you discharge. Oh, even the wound care instructions, [that] information may not sink in for the client because it is presented so fast and there may not be [time] for questions. Yeah, there are so many corners we cut. [….] …at a clinic last year, I saw them hold that yellow form [medical files] and going through [and just] check, check, check… adding blood pressure, adding all these things, just filling them in [without actually measuring blood pressure, etc.]. [Provider 3].

One assistant surgeon, a 25-year-old female nurse, claimed her record time for completing a circumcision was six minutes. She too associated VMMC targets with client overload, and with rushing surgeries and an increase in adverse events. She explained:

Sometimes you compete cutting clients and the clients are too much. The average time for a client’s [circumcision] is between 12 to 20 minutes. So when the clients are too much, and you want to meet your targets, you will perform the surgery a bit fast. So upon that you will have [an] adverse event and maybe a child will come back with a bleeder [an oozing artery or other blood vessel] you did not close [suture] well [Provider 4].

Despite these concerns, it was beyond the scope of our study to determine whether an increase in major or minor adverse events was associated with high volume periods. This issue should be addressed by future research.[38]

### Target alternatives

When asked about possible improvements to VMMC implementation and potential alternatives to the use of targets, interviewees made two suggestions. The first was to move away from active VMMC mobilization toward more passive strategies like those used by sites that provide walk-in HIV testing services in Kenya. The second was to keep donor targets, but to lower them to make them more possible to achieve. As Mobilizer 1 put it, “[VMMC] should be a walk-in thing where people are circumcised at their time of convenience…. Basically, I [don’t] have problems with targets, but what I have issues with is the extremely large targets. A situation where the surgeon strains to meet the target. And I know when the surgeon is strained, the probability of having adverse events is high.” Citing a similar alternative, as well as the implementation of more reasonable targets, Provider 1 offered:

The targets will dilute VMMC as a practice because the number is overwhelming. Imagine if you start working at 8.00am and you want to do circumcision [until] around 10 pm… it is a long day. So targets should be at least reasonable. So from my side, I can talk about the targets, and the targets [should be] revised …okay. Another thing is for instance… it should be incorporated to be like a clinic, in that if I come to the hospital I can even walk into the VMMC clinic. Not that I am being coerced to go for circumcision, but it should be part of us [an integrated part of the healthcare system]. You know the issue of targets is forcing people to go around looking for (clients) per day. It should be like a TB clinic. Yeah, like a walk-in clinic. So anybody can just come at his own time.

Yet another provider, when asked about the effects of targets on mobilizers, made comments that suggest the importance of improved communication as a means to decrease target burden on mobilizers for the benefit of all: “They [mobilizers] are a bit intimidated [by high targets]. So when they [VMMC technical advisors, program coordinators, etc.] set targets without consulting them, sometimes they even go slow and yeah, it affects our performance [numbers of clients to be circumcised]” [Provider 4].

## Discussion

Much effort has been made in epidemiological modelling of VMMC to determine who, when, and where to circumcise, and how many circumcisions will be necessary in order to curb the ESA HIV epidemic.[48–54] Yet in South Africa and elsewhere, studies have indicated that the pursuit of targets (for uptake of combination antiretroviral therapy) may be at odds with quality of care.[55] This study identifies multiple unintended consequences linked directly or indirectly to the use of targets for VMMC services in Kenya. As detailed above, these include pressures associated with VMMC client availability, extra burdening of facilities, overworked clinical staff, deviations from the standard of care, and long waits at the clinic.

In 2008, soon after the randomized controlled trials on HIV and male circumcision, and in advance of global efforts to implement VMMC, UNAIDS issued guidance for program decision-makers on human rights, ethics, and legal considerations.[56] This guidance advocates for a human rights based approach to the implementation of VMMC programs that ensures that the procedure is carried out safely, under conditions of informed consent, and without coercion or discrimination. On this basis, the UNAIDS guidance also explicitly states that, "target numbers of procedures, incentives to men, and incentives to providers should be avoided."[56] Similarly, PEPFAR guidelines have cautioned IPs against using targets to motivate mobilization or tethering VMMC remuneration to the number of procedures performed in order to avoid coercion.[57] According to the latest version of these guidelines, PEPFAR-funded IPs who reward mobilizers are:

…required to reward a team of mobilizers… so that any reward is based upon collective (versus individual) success. [This] approach limits the likelihood of coercion by separating any immediacy of reward resulting from an individual mobilizer referring a particular client. Mechanisms that further minimize perceived or actual rewards on a per-client/per-mobilizer basis are encouraged.[12]

Despite these guidelines, our data suggest that this stated preference for team rather than individual targets may be misplaced, or at least may not make as much of a difference as intended, since teams may still be incentivized to use misleading or otherwise questionable practices. While individual mobilizers may not receive an immediate payment on a per-client basis, they may be remunerated individually once they collectively (as a team) meet or surpass a given target. By coupling target achievement to remuneration, targets provide pressure to ensure that adequate numbers of adolescent circumcisions take place. Yet perversely, target pressure sometimes leads mobilization teams and providers to choose ethically dubious, but rational strategies to increase uptake, reduce effort, and/or shorten circumcision times to accomplish more MCs per day. Unfortunately, such strategies carry potentially deleterious outcomes for the boys this intervention is intended to protect. Moreover, they may not be in keeping with the ethics and human rights values to which VMMC funders and IPs purport to adhere.

Such strategies may include the mobilization of preadolescent clients, imbalanced discussions of benefits versus risks, abusive language and exploitation of social pressure to drive demand, and even dangerous shortcuts (e.g., rushing surgeries). One solution might involve increasing efforts to ensure that target achievement is entirely uncoupled from staff remuneration: mobilizers could draw salaries like the community health workers already working in the same communities—salaries that do not depend on targets. However, future research is needed to determine whether target alternative programs, such as the integrated healthcare/walk-in clinic/passive models suggested by our interviewees, can achieve the necessary number of MCs and do so without overly increasing costs. Indeed, efforts in Zimbabwe to integrate VMMC services into routine healthcare are encouraging,[58] and endeavors to integrate VMMC services in Kenya are already planned.[16] Yet, the performance based financial incentives used in Zimbabwe have been shown to be problematic[59] and our data suggest that this model may lead to similar issues in Kenya.

Concerning mobilizer messaging, others have previously criticized the PEPFAR-endorsed tactic of claiming as a “key message”[57] the benefit of a “60% reduction” in HIV risk.[60–64] During observations of VMMC mobilization activities in our study area, it became clear that few mobilizers and adolescent clients truly understood what the cited 60% reduction statistic would mean for them. More often, adolescents seemed confused and asked mobilizers to explain the “missing 40%”. Although some researchers have suggested moving beyond VMMC mobilization messages that emphasize HIV risk reduction to those that focus on a spectrum of benefits, including “hygiene, appearance, attractiveness to partners, peer norms, and modernity”,[65] our data suggest that mobilization strategies that rely on peer pressure may be equally problematic. For instance, is it ethical (or accurate) to suggest that to be circumcised is to be “modern” when less than 40% of men globally are circumcised and only a few high-income countries, including the US, have relatively high rates of MC [66, 67] (and even in the US, rates of MC have been falling)?[68–70] While it may be standard practice in health communication to describe relative versus absolute reductions in risk, especially when the baseline risk is relatively low or highly variable, VMMC mobilization efforts may represent a context in which departing from standard practice may be advisable. This is because the figure used (60%) is likely to foster an inflated view of the partial protection provided by MC to the individual, which may undermine informed consent. While we recognize that the promotional skits used during VMMC “roadshows” use humor and dramatize promotional messages to accentuate the positive, greater efforts should be made to ensure that this messaging is balanced so that benefits and risks are neither over- or understated. For similar reasons, the practice of public health workers persuading boys to become circumcised by claiming that their future female partners will definitely prefer them that way (and reject them if they are not) [45] should be questioned if not discouraged. In an effort to improve VMMC messaging, future studies could test the effectiveness of providing mobilizers with training in ethical conduct, as well as on the benefits versus risks of MC and how fairly and effectively to communicate this information to adolescents.

The practice of using in-school health talks for VMMC mobilization warrants special ethical scrutiny. Not only do teachers and other school authorities often attend these talks—individuals who may have undue influence over boys’ circumcision decisions—but the group-based nature of the talks, and the openness with which adolescent circumcision status is discussed and mobilization takes place, make these environments ripe for coercion. Recruitment of students for research in schools by teachers and other school authorities is discouraged by ethicists for similar reasons.[71] One possible solution might be to separate health talks from VMMC recruitment: mobilizers could conduct health talks one day and schedule a day to return. In a private setting on this second day, mobilizers could answer boys’ questions, discuss consent and assent, and take down the details of any boy who would like to proceed with MC.

In January 2019, our research team presented study findings to policy professionals and program officials at a VMMC stakeholder meeting in Kenya. In the discussion that followed, we considered various solutions to the ethical and practical concerns highlighted here. One strategy we discussed to address the problem of high and low seasons of client availability and improve the quality of care provided at VMMC clinics was for funders and IPs to collaborate to develop “smart targets.” These targets could be set to rise and fall with low and high seasons to match more efficiently resources and mobilization efforts to client demand and availability. As a part of this process, IP technical advisors could also work closely with school administrators in their catchment areas to find ways to conduct more MCs throughout the school year while minimizing the impact on educational activities and progress. Stakeholders also recognized that the training and awareness of mobilizers, counsellors, and clinic staff needs to be strengthened, and that quality assurance measures should be enhanced and better monitored. Future studies should test the cost and feasibility of improving these aspects of care under real-world conditions.

### Limitations

This is a qualitative study with very limited generalizability. Although we used purposive or convenience sampling methods in this study, efforts were made to select facilities of various sizes (levels 2–5), and to work equally with VMMC clinical staff and mobilization teams from both IP organizations. In addition, we conducted mobilization and clinic observation sessions during both the “high” and “low” VMMC seasons, including separate visits to a Level 3 and a Level 4 VMMC clinic on high and low volume days.

This study relied on in-depth interviews and clinic observations. As in most qualitative studies of this kind, interviewee responses could be subject to social desirability bias. While we cannot exclude entirely the possibility of this bias, we made clear that we were only interested in interviewees’ honest recollections of, and reflections on, their VMMC experiences. Moreover, the fact that interviewees were often critical of VMMC practices and, for instance, in the case of some clinical providers, admitted to cutting corners in care, suggests that this type of bias may be less of an issue in this study.

Finally, our presence as observers at VMMC facilities and during mobilization activities may have changed the behavior of staff members and clients. We anticipated this possibility early on and in response, chose a two-pronged approach method (i.e., in-depth interviews and observations) to ensure the quality of data. This potential bias was also considered during data analysis and presentation for publication. For instance, all findings based on observations were cross-verified by data from in-depth interviews, and vice versa.

## Conclusion

When VMMC for HIV prevention first began to be implemented in ESA, much attention was paid to the ethical, human rights, and legal considerations by the organizations that were (and are) involved in it. Since then, much of the discourse has been replaced by programmatic and technical guidance concerning how best to meet ambitious VMMC targets, with less attention to the broader effects of pursuing targets in the field. As our research shows, those considerations have not gone away. VMMC funders, governmental organizations, and IPs remain strongly committed to human rights and ethical values in the pursuit of public health goals. As such, they have a robust interest in information about how VMMC programs are implemented on the ground in Kenya and across ESA. These ethical considerations should be given more weight in future research and in the routine quality assurance checks conducted by IPs and funders, and where feasible, by independent authorities.

Further research is required to consider more fully the benefits versus risks associated with targets in mobilization activities and to develop improved or alternative systems through which to drive VMMC uptake. While PEPFAR already monitors VMMC clinical standards and practices (including recordkeeping) through the Site Improvement through Monitoring (SIMs) scheme,[12] future partnerships between funders, IPs, and local civil society organizations could be fostered or strengthened to avoid the unintended consequences noted above and to ensure that clients receive the best of available care. At a minimum, funders and governmental authorities should take on more target-setting input from IPs, including mobilizers and other staff on the ground, in an iterative and ongoing way. While it may not be reasonable or wise to call for the complete abandonment of the use of targets, given their necessity to intervention modelling and program budgeting, it is justified to be more critical of their use and aware of their potential unintended consequences.

## Supporting information

S1 File Stakeholder interview guides. (DOCX)

S2 File Government of Kenya voluntary medical male circumcision consent form. (PDF)

## References

[1] Weiss HA. Male circumcision as a preventive measure against HIV and other sexually transmitted diseases. Current Opinion In Infectious Diseases. 2007;20(1):66–72. 10.1097/QCO.0b013e328011ab73 . 17197884

[2] Sharma SC, Raison N, Khan S, Shabbir M, Dasgupta P, Ahmed K. Male circumcision for the prevention of human immunodeficiency virus (HIV) acquisition: a meta‐analysis. BJU International. 2018;121(4):515–26. 10.1111/bju.14102 . 29232046

[3] Fink AJ. A possible explanation for heterosexual male infection with AIDS. The New England journal of medicine. 1986;315(18):1167–. 3762636

[4] Reed JB, Njeuhmeli E, Thomas AG, Thomas AG, Bacon MC, Bailey R, et al Voluntary Medical Male Circumcision: An HIV Prevention Priority for PEPFAR. Journal of Acquired Immune Deficiency Syndromes. 2012;60(Suppl 3):S88–S95. 2012-20414-003. 22797745 10.1097/QAI.0b013e31825cac4e PMC3663585

[5] Hines JZ, Ntsuape OC, Malaba K, Zegeye T, Serrem K, Odoyo-June E, et al Scale-Up of Voluntary Medical Male Circumcision Services for HIV Prevention—12 Countries in Southern and Eastern Africa, 2013–2016. MMWR Morbidity And Mortality Weekly Report. 2017;66(47):1285–90. 10.15585/mmwr.mm6647a2 . 29190263 PMC5708689

[6] U.S. President's Emergency Plan for AIDS Relief (PEPFAR). PEPFAR Panorama Spotlight Washington, D.C.: PEPFAR; 2019 [cited 2019 11 June]. Available from: https://data.pepfar.gov/.

[7] U.S. President's Emergency Plan for AIDS Relief (PEPFAR). Kenya Country Operational Plan (COP/ROP) 2018 Strategic Direction Summary. Washington, D.C.: 2018.

[8] Mwandi Z, Murphy A, Reed J, Chesang K, Njeuhmeli E, Agot K, et al Voluntary medical male circumcision: translating research into the rapid expansion of services in Kenya, 2008–2011. Plos Medicine. 2011;8(11):e1001130–e. 10.1371/journal.pmed.1001130 . 22140365 PMC3226459

[9] U.S. President's Emergency Plan for AIDS Relief (PEPFAR). PEPFAR 2018 Annual Report to Congress. Washington, D.C.: PEPFAR, 2018.

[10] U.S. President's Emergency Plan for AIDS Relief (PEPFAR). PEPFAR Country/Regional Operational Plan (COP/ROP) Guidance 2017. Washington, D.C.: U.S. Department of State, 2017 18 January 2017. Report No.

[11] U.S. President's Emergency Plan for AIDS Relief (PEPFAR). PEPFAR 2018 Country Operational Plan Guidance for Standard Process Countries. Washington, D.C.: 2018.

[12] U.S. President's Emergency Plan for AIDS Relief (PEPFAR). PEPFAR's best practices for voluntary medical male circumcision site operations: A service guide for site operations. 2nd ed Washington, D.C.: PEPFAR (U.S. President's Emergency Plan for AIDS Relief); 2017.

[13] Davis SM, Hines JZ, Habel M, Grund JM, Ridzon R, Baack B, et al Progress in voluntary medical male circumcision for HIV prevention supported by the US President’s Emergency Plan for AIDS Relief through 2017: longitudinal and recent cross-sectional programme data. BMJ open. 2018;8(8):e021835 10.1136/bmjopen-2018-021835 30173159 PMC6120649

[14] U.S. President's Emergency Plan for AIDS Relief (PEPFAR). Kenya Country Operational Plan (COP) 2017: Strategic Direction Summary. Washington, D.C.: US Department of Health and Human Services; 2017 21 April 2017. Report No.

[15] Kripke K, Opuni M, Odoyo-June E, Onyango M, Young P, Serrem K, et al Data triangulation to estimate age-specific coverage of voluntary medical male circumcision for HIV prevention in four Kenyan counties. PloS one. 2018;13(12):e0209385 10.1371/journal.pone.0209385 30562394 PMC6298728

[16] National AIDS and STI Control Program (NASCOP). National Voluntary Medical Male Circumcision Strategy, 2014/15–2018/19. Nairobi: Government of Kenya, 2015.

[17] Rennie S, Muula AS, Westreich D. Male circumcision and HIV prevention: ethical, medical and public health tradeoffs in low-income countries. Journal Of Medical Ethics. 2007;33(6):357–61. 10.1136/jme.2006.019901 . 17526688 PMC2598273

[18] Luseno WK, Field SH, Iritani BJ, Rennie S, Gilbertson A, Odongo FS, et al Consent Challenges and Psychosocial Distress in the Scale-up of Voluntary Medical Male Circumcision Among Adolescents in Western Kenya. AIDS and behavior. 2019 10.1007/s10461-019-02620-7 31375957 PMC6854308

[19] Friedland BA, Apicella L, Schenk KD, Sheehy M, Hewett PC. How informed are clients who consent? A mixed-method evaluation of comprehension among clients of male circumcision services in Zambia and Swaziland. AIDS And Behavior. 2013;17(6):2269–82. 10.1007/s10461-013-0424-1 . 23392912

[20] Schenk K, Friedland B, Apicella L, Sheehy M, Munjile K, Hewett P. On the cutting edge: Improving the informed consent process for adolescents in Zambia undergoing male circumcision for HIV prevention. Vulnerable Children and Youth Studies. 2012;7(2):116–27. 10.1016/j.fitote.2016.06.018 PubMed PMID: 27370100.

[21] Schenk KD, Friedland BA, Sheehy M, Apicella L, Hewett PC. Making the cut: Evidence-based lessons for improving the informed consent process for voluntary medical male circumcision in Swaziland and Zambia. AIDS Education and Prevention. 2014;26(2):170–84. 10.1521/aeap.2014.26.2.170 2014-13016-007. 24694330

[22] Kaufman MR, Dam KH, Van Lith LM, Hatzold K, Mavhu W, Kahabuka C, et al Voluntary medical male circumcision among adolescents: a missed opportunity for HIV behavioral interventions. AIDS (London, England). 2017;31 Suppl 3:S233–S41. 10.1097/QAD.0000000000001484 . 28665881 PMC5497778

[23] Kaufman MR, Patel EU, Dam KH, Packman ZR, Van Lith LM, Hatzold K, et al Counseling Received by Adolescents Undergoing Voluntary Medical Male Circumcision: Moving Toward Age-Equitable Comprehensive Human Immunodeficiency Virus Prevention Measures. Clinical Infectious Diseases. 2018;66:S213–S20. 10.1093/cid/cix952 . 29617776 PMC5889033

[24] Kaufman MR, Patel EU, Dam KH, Packman ZR, Van Lith LM, Hatzold K, et al Impact of Counseling Received by Adolescents Undergoing Voluntary Medical Male Circumcision on Knowledge and Sexual Intentions. Clinical Infectious Diseases. 2018;66:S221–S8. 10.1093/cid/cix973 . 29617781 PMC5888933

[25] Jennings L, Bertrand J, Rech D, Harvey SA, Hatzold K, Samkange CA, et al Quality of voluntary medical male circumcision services during scale-up: a comparative process evaluation in Kenya, South Africa, Tanzania and Zimbabwe. Plos One. 2014;9(5):e79524–e. 10.1371/journal.pone.0079524 . 24801073 PMC4011679

[26] University Research Company. Potential Solutions to Common Quality Gaps in VMMC Programs. Maryland: USAID Appying Science to Strengthen and Improve Systems Project, 2017.

[27] National AIDS Control Council (NACC) and National AIDS and STIs Control Programme (NASCOP). Kenya HIV Estimates Report 2018. Nairobi: Kenya Ministry of Health, 2018.

[28] Muga R, Kizito P, Mbayah M, Gakuruh T. Overview of the health system in Kenya. Kenya service provision assessment (KSPA 2004) survey URL: https://dhsprogram com/pubs/pdf/spa8/02chapter2 pdf [accessed 2018-03-20][WebCite Cache ID 6y3kFHBkt]. 2005.

[29] Kimathi L. Challenges of the Devolved Health Sector in Kenya: Teething Problems or Systemic Contradictions? Africa Development. 2017;42(1):55–77.

[30] Bradley EH, Curry LA, Devers KJ. Qualitative data analysis for health services research: developing taxonomy, themes, and theory. Health services research. 2007;42(4):1758–72. Epub 2007/02/09. 10.1111/j.1475-6773.2006.00684.x 17286625 PMC1955280

[31] VERBI Software. MAXQDA 12. Berlin 2016.

[32] Frade S, Rech D, Spyrelis A, Machaku M, Mavhu W, Omondi D, et al. Seasonal patterns in voluntary medical male circumcision (VMMC) in South Africa, Kenya, Tanzania and Zimbabwe. 6th South African AIDS Conference; 18–21 June; Durban2013.

[33] Gold E, Mahler H, Boyee D. Overcoming seasonality in scaling up voluntary medical male circumcision. A case study from Tanzania. 2015.

[34] Macklin R. On paying money to research subjects: 'due' and 'undue' inducements. IRB: Ethics & Human Research. 1981;3(5):1–6. Epub 1981/05/01. . 11649367

[35] Curran K, Njeuhmeli E, Mirelman A, Dickson K, Adamu T, Cherutich P, et al Voluntary Medical Male Circumcision: Strategies for Meeting the Human Resource Needs of Scale-Up in Southern and Eastern Africa. PLOS Medicine. 2011;8(11):e1001129 10.1371/journal.pmed.1001129 22140364 PMC3226463

[36] Government of Kenya National AIDS and STI Control Program (NASCOP). Voluntary Medical Male Circumcision in Kenya: Report of the First Rapid Results Initiative conducted in November/December 2009. Nairobi: NASCOP, 2010 June. Report No.

[37] Herman-Roloff A, Bailey RC, Agot K. Factors associated with the safety of voluntary medical male circumcision in Nyanza province, Kenya. Bulletin of the World Health Organization. 2012;90(10):773–81. 10.2471/BLT.12.106112 . 23109745 PMC3471059

[38] Manentsa M, Mukudu H, Koloane N, Ringane A, Matta E, Martinson NA, et al Complications of high volume circumcision: glans amputation in adolescents; a case report. BMC Urolology. 2019;19(1):65 Epub 2019/07/13. 10.1186/s12894-019-0462-8 31296191 PMC6625076

[39] Gray R, Kigozi G, Serwadda D, Makumbi F, Watya, et al Male circumcision for HIV prevention in men in Rakai, Uganda. Lancet. 2007;369(9562):657–66. 10.1016/S0140-6736(07)60313-4 17321311

[40] Bailey R, Moses S, Parker C, Agot K, Maclean I, et al Male circumcision for HIV prevention in young men in Kisumu, Kenya: a randomised controlled trial. Lancet. 2007;369(9562):643–56. 10.1016/S0140-6736(07)60312-2 17321310

[41] Auvert B, Taljaard D, Lagarde E, Sobngwi-Tambekou J, Sitta R, et al Randomized, controlled intervention trial of male circumcision for reduction of HIV infection risk: the ANRS 1265 trial. PLoS medicine. 2005;3(5):e226. 10.1371/journal.pmed.0020298 PMC1262556 16231970

[42] Gray R, Kigozi G, Kong X, Ssempiija V, Makumbi F, Wattya S, et al The effectiveness of male circumcision for HIV prevention and effects on risk behaviors in a posttrial follow-up study. AIDS (London, England). 2012;26(5):609–15. Epub 2012/01/03. 10.1097/QAD.0b013e3283504a3f 22210632 PMC4296667

[43] Auvert B, Taljaard D, Rech D, Lissouba P, Singh B, Shabangu D, et al., editors. Effect of the Orange Farm (South Africa) male circumcision roll-out (ANRS-12126) on the spread of HIV. 6th IAS Conference on HIV Pathogenesis, Treatment and Prevention; 2011.

[44] Riess TH, Achieng MM, Bailey RC. Women's beliefs about male circumcision, HIV prevention, and sexual behaviors in Kisumu, Kenya. PloS one. 2014;9(5):e97748–e. 10.1371/journal.pone.0097748 . 24844845 PMC4028254

[45] Osaki H, Mshana G, Wambura M, Grund J, Neke N, Kuringe E, et al 'If you are not circumcised, I cannot say yes': The role of women in promoting the uptake of voluntary medical male circumcision in Tanzania. PloS one. 2015;10(9). 2016-03432-001. 10.1371/journal.pone.0139009 PMC4581795 26402231

[46] Kaufman MR, Dam KH, Sharma K, Van Lith LM, Hatzold K, Marcell AV, et al Females' Peer Influence and Support for Adolescent Males Receiving Voluntary Medical Male Circumcision Services. Clinical Infectious Diseases. 2018;66:S183–S8. 10.1093/cid/cix1057 . 29617773 PMC5888916

[47] Morris BJ, Hankins CA, Banerjee J, Lumbers ER, Mindel A, Klausner JD, et al Does Male Circumcision Reduce Women's Risk of Sexually Transmitted Infections, Cervical Cancer, and Associated Conditions? Frontiers in Public Health. 2019;7. 10.3389/fpubh.2019.00004 PMC6365441 30766863

[48] Njeuhmeli E, Forsythe S, Reed J, Opuni M, Bollinger L, Heard N, et al Voluntary medical male circumcision: modeling the impact and cost of expanding male circumcision for HIV prevention in eastern and southern Africa. PLoS medicine. 2011;8(11):e1001132 10.1371/journal.pmed.1001132 22140367 PMC3226464

[49] Blaizot S, Maman D, Riche B, Mukui I, Kirubi B, Ecochard R, et al Potential impact of multiple interventions on HIV incidence in a hyperendemic region in Western Kenya: a modelling study. BMC Infectious Diseases. 2016;16(1):189 10.1186/s12879-016-1520-4 27129591 PMC4851795

[50] Hankins C, Warren M, Njeuhmeli E. Voluntary Medical Male Circumcision for HIV Prevention: New Mathematical Models for Strategic Demand Creation Prioritizing Subpopulations by Age and Geography. PloS one. 2016;11(10):e0160699–e. 10.1371/journal.pone.0160699 . 27783613 PMC5082625

[51] Kripke K, Chimbwandira F, Mwandi Z, Matchere F, Schnure M, Reed J, et al Voluntary Medical Male Circumcision for HIV Prevention in Malawi: Modeling the Impact and Cost of Focusing the Program by Client Age and Geography. PloS one. 2016;11(7):1–11. 10.1371/journal.pone.0156521 . PMC4943664 27410474

[52] Kripke K, Okello V, Maziya V, Benzerga W, Mirira M, Gold E, et al Voluntary Medical Male Circumcision for HIV Prevention in Swaziland: Modeling the Impact of Age Targeting. PLOS ONE. 2016;11(7):e0156776 10.1371/journal.pone.0156776 27410687 PMC4943626

[53] Kripke K, Vazzano A, Kirungi W, Musinguzi J, Opio A, Ssempebwa R, et al Modeling the Impact of Uganda’s Safe Male Circumcision Program: Implications for Age and Regional Targeting. PloS one. 2016;11(7):e0158693 10.1371/journal.pone.0158693 27410234 PMC4943628

[54] McGillen JB, Stover J, Klein DJ, Xaba S, Ncube G, Mhangara M, et al The emerging health impact of voluntary medical male circumcision in Zimbabwe: An evaluation using three epidemiological models. PloS one. 2018;13(7):e0199453 10.1371/journal.pone.0199453 30020940 PMC6051576

[55] de Kok BC, Widdicombe S, Pilnick A, Laurier E. Doing patient-centredness versus achieving public health targets: A critical review of interactional dilemmas in ART adherence support. Social Science & Medicine. 2018;205:17–25. 10.1016/j.socscimed.2018.03.030. 29631198

[56] Joint United Nations Programme on HIV/AIDS (UNAIDS). Safe, Voluntary, Informed Male Circumcision and Comprehensive HIV Prevention Programming: Guidance for decision-makers on human rights, ethical and legal considerations. Geneva: UNAIDS, 2008 June 2007. Report No.

[57] U.S. President's Emergency Plan for AIDS Relief (PEPFAR). PEPFAR best practices for voluntary medical male circumcision site operations: A service guide for site operations. PEPFAR Washington, DC; 2013.

[58] Feldacker C, Makunike-Chikwinya B, Holec M, Bochner AF, Stepaniak A, Nyanga R, et al Implementing voluntary medical male circumcision using an innovative, integrated, health systems approach: experiences from 21 districts in Zimbabwe. Global health action. 2018;11(1):1414997–. 10.1080/16549716.2017.1414997 . 29322867 PMC5769777

[59] Feldacker C, Bochner AF, Herman-Roloff A, Holec M, Murenje V, Stepaniak A, et al Is it all about the money? A qualitative exploration of the effects of performance-based financial incentives on Zimbabwe's voluntary male medical circumcision program. PloS one. 2017;12(3):1–15. 10.1371/journal.pone.0174047 . PMC5354455 28301588

[60] Masukume G. The ethics of claiming a 60% reduction in HIV acquisition from voluntary medical male circumcision. South African Journal of Bioethics and Law. 2014;7(1):4–.

[61] Green LW, Travis JW, McAllister RG, Peterson KW, Vardanyan AN, Craig A. Male circumcision and HIV prevention: Insufficient evidence and neglected external validity. American journal of preventive medicine. 2010;39(5):479–82. 10.1016/j.amepre.2010.07.010 20965388

[62] Gwandure C. The ethical concerns of using medical male circumcision in HIV prevention in Sub-Saharan Africa. South African Journal of Bioethics and Law. 2011;4(2):89–94.

[63] Van Howe RS, Storms MR. How the circumcision solution in Africa will increase HIV infections. Journal of public health in Africa. 2011;2(1):e4–e. 10.4081/jphia.2011.e4 . 28299046 PMC5345479

[64] Svoboda JS, Adler PW, Van Howe RS. Circumcision Is Unethical and Unlawful. The Journal Of Law, Medicine & Ethics: A Journal Of The American Society Of Law, Medicine & Ethics. 2016;44(2):263–82. 10.1177/1073110516654120 . 27338602

[65] Sgaier SK, Reed JB, Thomas A, Njeuhmeli E. Achieving the HIV Prevention Impact of Voluntary Medical Male Circumcision: Lessons and Challenges for Managing Programs. PLoS medicine. 2014;11(5):e1001641 10.1371/journal.pmed.1001641 24800840 PMC4011573

[66] Morris BJ, Wamai RG, Henebeng EB, Tobian AA, Klausner JD, Banerjee J, et al Estimation of country-specific and global prevalence of male circumcision. Population Health Metrics. 2016;14(1):4 10.1186/s12963-016-0073-5 26933388 PMC4772313

[67] Weiss H, Polonsky J, Bailey R, Hankins C, Halperin D, Schmid G. Male circumcision: global trends and determinants of prevalence, safety and acceptability. World Health Organization and the Joint United Nations Programme on HIV/AIDS (UNAIDS) 2007.

[68] Owings M, Uddin S, Williams S. Trends in circumcision for male newborns in US hospitals. NCHS health notes: Citeseer; 2013.

[69] Morris BJ, Bailis SA, Wiswell TE, editors. Circumcision rates in the United States: rising or falling? What effect might the new affirmative pediatric policy statement have? Mayo Clinic Proceedings; 2014: Elsevier. 10.1016/j.mayocp.2014.01.001 24702735

[70] Mor Z, Kent CK, Kohn RP, Klausner JD. Declining Rates in Male Circumcision amidst Increasing Evidence of its Public Health Benefit. PloS one. 2007;2(9):e861 10.1371/journal.pone.0000861 17848992 PMC1955830

[71] Bonham VH, Morenso JD. Research with captive populations: Prisoners, students, and soldiers In: Emanuel EJ, Grady CC, Crouch RA, Lie RK, Miller FG, Wendler DD, editors. The Oxford Handbook of Clinical Research Ethics. Oxford: Oxford University Press; 2008.

