# Supplementary material for: Voluntary medical male circumcision for HIV prevention among adolescents in Kenya: Unintended consequences of pursuing service-delivery targets
Source: PLoS One. 2019 Nov 4;14(11):e0224548. doi: 10.1371/journal.pone.0224548 (PMC6827911; doi:10.1371/journal.pone.0224548)
Supplement: S1 File — (DOCX) [file pone.0224548.s001.docx]

**S1. Stakeholder interview guides.**

**Interview Guide for Teachers for VMMC Study**

October 4, 2016

**Introduction:** I am going to ask you some questions about your experiences with medical male circumcision in regard to your school, and your opinions about it. Please remember there is no right or wrong answer to these questions. I just want to hear your answers in your own words from your own perspective. Please be assured that any information you give me is confidential and only authorized research staff will have access to anything you share with me. Also, you may skip any question that you feel uncomfortable answering. Do you have any questions before we begin?

Thank you for agreeing to participate in this interview. Before we begin the interview, I am going to collect some basic information about you.

Demographic questions:

1. Date of interview
2. Participant ID
3. Name of interviewer
4. Village name
5. Name of school
6. Job title (e.g, headmaster, deputy headmaster, teacher, counselor, other)
7. DOB
8. Age at last birthday
9. Gender
10. Highest education level completed
11. Polytechnic
12. Religion
13. Ethnicity
14. Position

I will now turn on the audio recorder and begin the interview.

**Interviewer turns on the audio-recorder to begin interview. Note taking is done using the assigned unique ID.**

1. How long have you been teaching (or whatever they do at the school)? What subjects do you teach? Did you grow up in the area? Where did you train as a teacher? How long have you been working at this school?
2. Could you please tell me about your experience with VMMC at your school?
   1. *Are parents brought into the school to learn about VMMC? If so, what information are they given and by whom?*
   2. *Do children bring home information (e.g., information sheets/handouts) on VMMC? If so, what handouts are provided? Who designs these handouts? Do you have an example I could have?*
   3. *How do VMMC activities fit into the normal school schedule?*
3. Could you tell me specifically when you were contacted about VMMC mobilization and what specific tasks you were asked to do?
   1. *What were your first thoughts or impressions concerning VMMC?*
   2. *What are your personal feelings towards VMMC? Do you support VMMC?*
   3. *Do you or your school receive any incentives for helping to recruit students for VMMC? If so, what are the incentives?*
   4. *What were the approximate dates for each time you were contacted about VMMC mobilization?*
   5. *What, if any, training were you given to take on these specific tasks? Who conducted this training?*
   6. *What happens in a scenario where a parent or adolescent is reluctant to take part in VMMC? Do you try to persuade them? Can you give me an example of a time when this happened?*
4. What specific information is given to adolescents and parents at the school about VMMC?
   1. *How is this information communicated (e.g., via media? If so, which media? Handout? In class?)*
   2. *If I were a male student in your school, what would I be told about VMMC?*
   3. *How have the students and their parents responded so far?*
5. What information is given to adolescents and parents at the school about HIV testing as part of VMMC?
6. Are you involved at all in the consent processes? If yes, please describe your involvement?
   1. IF INVOLVED: Can you please list the specific procedures by which you obtain informed consent?
      1. *Are you required to obtain parental consent for boys under 18 for VMMC?*
      2. *Please describe whether this is different for boys under 16 versus age 16-18.*
      3. *Say there is a case where a boy under 18 wants to be circumcised but does not have parental consent, what would happen?*
      4. *Under what specific circumstances could boys under 18 years old sign for themselves?*
      5. *Could you please describe how consent is obtained (e.g. verbal or written)? How is consent documented? Where is consent documentation kept?*
   2. IF NOT INVOLVED, who obtains informed consent? What are the procedures under which the person you stated obtains informed consent?
7. Have you experienced any problems because a boy was circumcised without parental consent? What about any problems where the parents consented but the boy did not assent? Could you please tell me more…?
8. How are children at the school who are interested in VMMC brought to be circumcised? (E.g. in a bus or other vehicle)
9. Could you please describe any feedback you have received about VMMC from parents?
   1. *Did you receive any negative feedback? (E.g. did some parents feel like they were not informed or not asked to consent? Minors being tested for HIV without a parent present?) Positive feedback?*
10. Could you please describe any feedback you have received about VMMC from adolescents?
    1. *Did you receive any negative feedback? Positive feedback?*
11. Could you please describe any feedback you have received about VMMC from other teachers?
    1. *Did you receive any negative feedback? Positive feedback?*

**Interview Guide for Mobilizers for VMMC Study**

**Introduction:** I am going to ask you some questions about your experiences with medical male circumcision and your opinions about it. Please remember there is no right or wrong answer to these questions. I just want to hear your answers in your own words from your own perspective. Please be assured that any information you give me is confidential and only authorized research staff will have access to anything you share with me. Also, you may skip any question that you feel uncomfortable answering. Do you have any questions before we begin?

Thank you for agreeing to participate in this interview. Before we begin the interview, I am going to collect some basic information about you.

Demographic questions:

- - - 1. Date of interview
      2. Participant ID
      3. Name of interviewer
      4. Village name
      5. Name of employer
      6. Job title
      7. DOB
      8. Age at last birthday
      9. Gender
      10. Highest education level completed
      11. Polytechnic
      12. Religion
      13. Ethnic affiliation

I will now turn on the audio recorder and begin the interview.

**Interviewer turns on the audio-recorder to begin interview. Note taking is done using the assigned unique ID.**

1. Tell me what you do as [job title]. What circumstances led you to taking the position of [job title] with [name of institution]? Have you worked with any other organization in a similar role? If so, which ones? Have you worked on health promotion projects before? In general, what have been your experiences working on the VMMC campaign/project?
2. How long have you been working for [name of institution] on the VMMC campaign/project? Tell me how you are paid for your services as a [job title] for [name of institution]? For example, are you paid a salary on a monthly basis or are you paid in another way? Which of these ranges would you say best estimates your monthly income as a [job title] for [name of institution]: KSh 0-20,000; Ksh 20,001-30,000; Ksh 30,001-40,000; Ksh 40,001-50,000?
3. In general, what was the training like for your current position?
   1. *How long was your training? Who conducted the training? Where was it held?*
4. What supervision do you receive in your role as mobilizer?
   1. *Who supervises you? How do they supervise you?*
5. During your training, how were you instructed to recruit clients for VMMC? How does recruitment of clients work in real life? Please explain.
6. Kindly describe the typical recruitment activities for VMMC.
   1. How do you recruit adolescents under age 18? *Please describe your role within each of these activities.* What do you like and dislike about each of these activities?
7. How does the role of mobilizer and counselor differ? Do you ever take on the role of counselor? If so, under what circumstances?
8. What would you say is the most challenging part of your job?
9. Please describe what venues you typically use to mobilize boys? What about to mobilize parents?
10. How do you feel about targets and quotas for recruitment?
    1. *Are there targets/quotas for recruitment? If so, what are the targets/quotas? Who sets the targets/quotas?*
    2. *Do mobilizers receive bonuses for meeting or exceeding quotas? Are these bonuses added to your salary or given to you separately?*
    3. *What would happen if a recruiter does not meet his/her target/quota?*
    4. *Could you provide an example of an occasion when colleague or yourself did not meet your target/quota and explain what happened?*
11. Let’s say I am a boy who is considering VMMC, what would you tell me?
    1. *What kinds of handouts do you provide?* *Who develops these materials? What is good about these materials? What could be improved about these materials? May I have a copy of what you provide?*
12. Now let’s say I am a parent of an adolescent interested in VMMC, what you would tell me?
13. Kindly share with me some of the quality assurance procedures put in place at your work place for VMMC. Are you always able to follow them? Please tell me why or why not.
    1. *How are you required to document them?*
    2. *How often do you submit these documents?*
14. Tell me about informed consent for VMMC with parents and boys.
    1. *Is it also part of your job as mobilizer/recruiter to obtain informed consent for VMMC?*
       1. IF YES. Could you please list the specific procedures by which you obtain informed consent?
          1. *Are you required to obtain parental consent for boys under 18 for VMMC? Is consent for HIV testing also included?*
          2. *Please describe whether this is different for boys under 16 versus age 16-18.*
          3. *Under what specific circumstances could boys under 18 years old sign for themselves?*
          4. *Could you please describe how consent is obtained (e.g. verbal or written)? How is consent documented? Where is consent documentation kept?*
          5. *What are the greatest challenges you have experienced related to informed consent?*
    2. IF NO, who obtains informed consent? What procedures does that person use?
15. I can imagine that some of the boys you talk to are reluctant to be circumcised. Do you try to change their minds? If so, how do you do that?
16. Say there is a case where a boy under 18 wants to be circumcised but does not have parental consent. Could you please explain what is supposed happen? Is this what tends to happen in real life?
17. Have you experienced any problems because a boy was circumcised without parental consent? What about any problems where the parents consented but the boy did not agree? Could you please tell me more…
18. Could you please describe the process by which boys are tested for HIV prior to VMMC? What do you think about testing in this way? What is good and bad about it?
    1. *Are parents asked to accompany boys for testing? If so, do the parents understand why this is taking place?*
    2. *What happens if the boy is found to be positive?*
       1. *Would he still be offered circumcision? Would he be referred for HIV services?*

**Interview Guide for Counselors for VMMC Study**

**Introduction:** I am going to ask you some questions about your experiences with medical male circumcision and your opinions about it. Please remember there is no right or wrong answer to these questions. I just want to hear your answers in your own words from your own perspective. Please be assured that any information you give me is confidential and only authorized research staff will have access to anything you share with me. Also, you may skip any question that you feel uncomfortable answering. Do you have any questions before we begin?

Thank you for agreeing to participate in this interview. Before we begin the interview, I am going to collect some basic information about you.

Demographic questions:

1. Date of interview
2. Participant ID
3. Name of interviewer
4. Village name
5. Name of employer
6. Job title
7. DOB
8. Age at last birthday
9. Gender
10. Highest education level completed
11. Polytechnic
12. Religion
13. Ethnic affiliation

I will now turn on the audio recorder and begin the interview.

**Interviewer turns on the audio-recorder to begin interview. Note taking is done using the assigned unique ID.**

1. Tell me what you do as [job title] for [name of institution]. What circumstances led you to taking the position of VMMC counselor? Have you worked with any other organization in a similar role? If so, which ones? Have you worked on health promotion campaigns/projects before? In general, what have been your experiences working on the VMMC campaign?
2. How long have you been working for [institution] on the VMMC campaign? Tell me how you are paid for your services as a [job title] for [name of institution]? For example, are you paid a salary on a monthly basis or are you paid in another way? [If they say another way, you want the interviewer to probe to find out how, e.g., whether it’s on a commission basis for completed circumcisions.] Which of these ranges would you say best estimates your monthly income as a [job title] for [name of institution]: KSh 0-20,000; Ksh 20,001-30,000; Ksh 30,001-40,000; Ksh 40,001-50,000?
3. Tell me about your job as a counsellor. Describe a typical day of work for you. How many clients do you see? What is your role in meeting with them? What do you like or dislike about your job?
4. What supervision do you receive in your role as counselor?
   1. *Who supervises you? How do they supervise you?*
5. What are the guidelines for recruitment for VMMC? Is this how it works in real life? Please explain.
6. Could you describe typical recruitment activities for VMMC?
   1. *Please describe your role within each of these activities.*
7. How are targets and quotas used for recruitment and counseling?
   1. *Are there targets/quotas for counseling? If so, what are the targets/quotas? Who sets the targets/quotas?*
   2. *Do counselors receive bonuses for meeting or exceeding quotas? Are these bonuses added to your salary or given to you separately?*
   3. *What would happen if a counselor does not meet his/her target/quota?*
   4. *Could you provide an example of an occasion when colleague or yourself did not meet your target/quota and explain what happened?*
8. Could you please describe the training that you had to complete for your current role? What was the required education and work experience for your current role?
9. What specific quality assurance procedures are in place or expected? Are you always able to follow them? Please tell me why or why not.
   1. What was the training like for your current position?
   2. *How long was your training? Who conducted the training? Where was it held?*
   3. *Are you required to document your work? If so, what must be documented?*
   4. *How often do you submit these documents and to whom? Are you ever given feedback about what you submit? What happens if you found a problem or something that is not covered in your training or guidelines? Do you receive support and help to make decisions?*
10. Let’s say I am a boy who is considering VMMC, what would you tell me?
    1. *What kinds of handouts do you provide?* *Who develops these materials? What is good about these materials? What could be improved about these materials? Could we have an example of them? [Ask for a full set of what is given to minors and parents.]*
11. Now let’s say I am a parent of an adolescent interested in VMMC, what would you tell me?
12. What role do you play in providing information and obtaining informed consent?
    1. Could you please list the specific procedures by which you obtain informed consent?
       1. *Are you required to obtain parental consent for boys under 18 for VMMC?*
       2. *Please describe whether this is different for boys under 16 versus age 16-18.*
       3. *Under what specific circumstances could boys under 18 years old sign for themselves?*
       4. *Could you please describe how permission from parents and agreement from adolescents are obtained (e.g. verbal or written)? How is consent documented? Where is consent documentation kept?*
       5. *What are parents and adolescents consenting for? Is it only for circumcision or also for other services? What other services? Do they understand this?*
       6. *What are the greatest challenges you have experienced related to informed consent?*
13. Say there is a case where a boy under 18 wants to be circumcised but does not have parental permission. Could you please explain what is supposed happen? Is this what tends to happen in real life?
14. Have you experienced any problems because a boy was circumcised without parental consent? What about any problems where the parents consented but the boy did not agree? Could you please tell me more…?
15. I can imagine that some of the boys you talk to are reluctant to be circumcised. Do you try to change their minds? If so, how do you do that?
16. Could you please describe the process by which boys are tested for HIV prior to VMMC? How do you feel about testing this way? What is good and bad about it?
    1. *Are parents asked to accompany boys for testing? Do the parents understand why this is done?*
    2. *What happens if the boy is found to be positive?*
       1. *Would he still be offered circumcision? Would he be referred for HIV services? Can you give me any examples of when this has happened and what occurred?* **Interview Guide for Providers for VMMC Study**

**Introduction:** I am going to ask you some questions about your experiences with medical male circumcision in regard to your patients, and your opinions about it. Please remember there is no right or wrong answer to these questions. I just want to hear your answers in your own words from your own perspective. Please be assured that any information you give me is confidential and only authorized research staff will have access to anything you share with me. Also, you may skip any question that you feel uncomfortable answering. Do you have any questions before we begin?

Thank you for agreeing to participate in this interview. Before we begin the interview, I am going to collect some basic information about you.

Demographic questions:

- - - 1. Date of interview
      2. Participant ID
      3. Name of interviewer
      4. Village name
      5. Name of institution
      6. Job title (e.g., in-charge, doctor, clinical officer (CO), registered nurse)
      7. DOB
      8. Age at last birthday
      9. Gender
      10. Highest education level completed
      11. Polytechnic
      12. Religion
      13. Ethnic affiliation

I will now turn on the audio recorder and begin the interview.

**Interviewer turns on the audio-recorder to begin interview. Note taking is done using the assigned unique ID.**

1. How long have you been a [job title]? Where were you trained? How long have you been working at [name of facility]?
2. How long have been doing VMCC? Did you do medical circumcision (MC) before, prior to VMMC for HIV prevention? Where were you trained to do MC/VMMC? Have you done MC/VMMC at other facilities besides [name of facility]?
3. How many circumcisions for VMMC do you do in a month? How many adults ages 18 and older? How many minors aged 14 through 17? How many children younger than 14?
4. For what percent of your time at work do you do circumcisions for VMMC? Who else does VMMC at [name of facility]? How do you share duties? Do you work as a team or individually to do the surgeries? Is there a special day each week when you do them or do you perform them on demand?
5. Outside of the surgical procedure, could you please describe any other roles you have for VMMC?
   1. For instance, do you have any role in recruitment, informed consent, or quality assurance?
   2. Do you sometimes assist with surgery or act as the infection control officer?
   3. Do you give input for VMMC policies?
   4. Have you acted as a consultant? [If yes, ask them to say more about this.]
6. Could you please describe what members of the team are present prior to, during, or after the circumcision procedure and their individual roles?
   1. *Is an infection prevention officer on the team? If so, what is his/her specific role and how do they act to prevent infections?*
7. Could you please describe the process for obtaining consent for MC? How is this different for boys under 18 years of age?
   1. *Is this different for those under 16 than for those 16-18?*
8. Are you often in communication with parents about the procedure? What are some of their common questions and concerns?
9. Could you please describe the process by which boys are tested for HIV prior to VMMC?
   1. *Who does the testing?*
   2. *Are you made aware of the results of a patient prior to the procedure?*
   3. *Are parents asked to accompany boys for testing?*
   4. *What happens if the boy is found to be positive? Is the procedure still performed? If so, why?*
10. How does the circumcision surgical procedure itself differ for boys under 18 compared to men?
11. Do most of the boys under 18 you circumcise have access to follow-up care? If so, who provides this care?
    1. *Let’s say I am a boy, what would you tell me about the procedure and follow-up care?*
12. In your experience, do adolescents come back for follow-up check-ups?
    1. *What are the reasons they may not come back? What happens if they don’t come for their check-ups? Are there infection problems?*
    2. *Does someone go out to check on them?*
    3. *Is their absence for follow-up documented? And is it monitored through the quality assurance process?*
    4. Have you seen or heard about any complications of the procedure? If so, what were they?
    5. Have you had parents or boys come to you to complain about the surgery?
13. Do you feel like you have enough help and support from the facility/county/program implementors to conduct safe MC and follow-up with patients? Why or why not? Please explain.
    1. Are there times when targets need to be met for MC? What do these targets mean for clinicians?
14. What specific quality assurance procedures do you follow? What, if any, challenges do you experience concerning these procedures?
    1. *How are you required to document them?*
    2. *How often do you submit these documents?*
    3. *What do you think about the quality assurance process?*
       1. *Do you feel like the documents you submit are read and acted upon?*
       2. *Has it resulted in any improvements to clinical care?*
    4. *Have there been any safety issues? If so, tell me about them.*
    5. *Have there been any shortages of supplies? If so, tell me about them.*
15. Are there any special issues for adolescents under 18 years of age? If yes, what are they?
    1. Wound reopening (possibly due to sexual activity)
    2. Consent? Parental permission?
    3. Resistance towards VMMC by the community?
    4. Lack of information or misinformation?
16. Do you have male children? Would you mind telling me if they have been circumcised and why you made this decision? [Tell them they don’t need to answer the question.]

**Interview Guide for Policy Makers for VMMC Study**

**Introduction:** I am going to ask you some questions about your experiences with medical male circumcision as an approach to HIV prevention in Kenya, and your opinions about it. Please remember there is no right or wrong answer to these questions. I just want to hear your answers in your own words from your own perspective. Please be assured that any information you give me is confidential and only authorized research staff will have access to anything you share with me. Also, you may skip any question that you feel uncomfortable answering. Do you have any questions before we begin?

Thank you for agreeing to participate in this interview. Before we begin the interview, I am going to collect some basic information about you.

Demographic questions:

1. Date of interview
2. Participant ID
3. Name of interviewer
4. Workstation
5. Name of institution
6. Job title (e.g., County Director of Health, KEMRI/CDC Technical Advisor)
7. DOB
8. Age at last birthday
9. Gender
10. Highest level of education

I will now turn on the audio recorder and begin the interview.

**Interviewer turns on the audio-recorder to begin interview. Note taking is done using the assigned unique ID.**

1. How long have you worked for [name of institution? Tell me about your position? How long have you worked in this position? How did you get into this type of work?
2. Of countries in Africa with VMMC programs, Kenya has circumcised more male adolescents than any other nation so far. Do you see this as a ‘success story’? If yes, why do you think it has been a success?
3. Is the VMMC policy to circumcise as many males as possible to help prevent HIV accepted by the community? Has there been any resistance towards it?
4. What are the important issues that must be considered concerning VMMC among minors (less than 18 years of age)?
5. Tell me about VMMC guidelines for minors in Kenya.
   1. *Are there standardized guidelines for VMMC in Kenya? If so, what are they?*
6. Could you describe any service delivery targets that are in place for VMMC?
   1. *Who sets the targets?*
   2. *How are the targets developed?*
   3. *How are the targets communicated to those in the field?*
   4. *What happens when targets are not met?*
   5. *What difficulties do counsellors and mobilizers face in meeting targets?*
7. Can you say more about the specific procedures by which informed consent is obtained?
   1. *Is parental consent required for boys who are under the age of 18? If so, what are the procedures by which parental consent is obtained?*
   2. *Under what circumstances, if any, is a boy under 18 allowed to consent for himself for VMMC in Kenya?*
   3. *Please describe whether this is different for boys under 16 versus age 16-18.*
   4. *Could you please describe how consent is obtained (e.g. verbal or written)? How is consent documented? Where is consent documentation kept? How is it compiled?*
   5. *From your experience, what are the greatest challenges concerning informed consent? What difficulties do counselors and mobilizers face in obtaining informed consent?*
8. Could you please describe the procedures for quality assurance for VMMC?
   1. Who conducts the quality assurance process?
   2. What do you think about the overall process of quality assurance?
      1. *Do you regularly review and use quality assurance data? If so, in what ways do you use the data? Do you know other policy makers who use QA information?*
      2. *Tell me about the people who conduct VMMC QA? What kinds of problems have they found with minors?*
   3. From your experiences, what are the greatest challenges concerning quality assurance?
   4. What are the procedures if problems are found during the quality assurance process?
   5. Could you tell me how complaints from the public are handled?
   6. How have you incorporated findings of quality assurance monitoring into implementation policies and practices?
9. What authorities have what roles when it comes to creating and implementing VMMC policy? Who is responsible for VMMC implementation in Siaya County?
   1. *Who authored the VMMC guidelines? Are they enforced in Siaya?*
   2. *What are the mechanisms of accountability?*
   3. *Could you tell me who plans and coordinates VMMC services at the county and sub-county levels?*
10. What are the specific mechanisms by which information about VMMC is communicated to target audiences?
11. Could you please describe the process by which boys are tested for HIV prior to VMMC?
    1. Do you think that there is anything that needs to be improved about this process?
    2. *Are parents asked to accompany boys for testing?*
    3. *What happens if the boy is found to be positive? Do you know of any challenges in disclosure or in linking minors with HIV services?*
12. If you were able to change some part of the current VMMC policy, what might you change?
