# Supplementary material for: Voluntary medical male circumcision for HIV prevention among adolescents in Kenya: Unintended consequences of pursuing service-delivery targets
Source: PLoS One. 2019 Nov 4;14(11):e0224548. doi: 10.1371/journal.pone.0224548 (PMC6827911; doi:10.1371/journal.pone.0224548)
Supplement: S2 File — (PDF) [file pone.0224548.s002.pdf]

REPUBLIC OF KENYA

MINISTRY OF HEALTH

Consent Form for Operation

I ______________________________ of P.O. Box ____________________ hereby consent myself/child/spouse/relative (name) ______________________________ to undergo the operation of ______________________________. The nature and possible complications of the operation and general/local anaesthesia has been explained to me fully by the clinician. I also consent to any further or alternative procedures to be performed, which the clinician may find necessary during the operation. I am not in knowledge of which clinician will perform the operation.

Patient's/Client's/parent's/guardian's sign: ____________________ Date: ____________

Doctor's sign: ____________________ Date: ____________________

Witness' sign: ____________________ Date: ____________________

REPUBLIC OF KENYA

MINISTRY OF HEALTH

Kibali cha Upasuaji

Mimi ______________________________ wa S.L.P. ____________________ natoa kibali changu/ kwa mtoto wangu/ bibi wangu ______________________________ afanyiwe upasuaji wa ______________________________ ambao maana yake na matokeo yake nimeelezwa na Daktari ____________________ Tena natoa kibali ili kutumiwe dawa ya kupoteza fahamu au upasuaji wowote atakaopatikana wa manufaa wakati wa shughuli hiyo na Daktari. Sijafahamishwa ni daktari yupi atakayefanya upasuaji.

Sahihi ya mteja/mzazi/mlezi: ____________________ Tarehe: ____________

Sahihi ya daktari: ____________________ Tarehe: ____________

Sahihi ya shahidi: ____________________ Tarehe: ____________

CIRCUMCISION INFORMATION

What is circumcision?

Circumcision is a minor surgical procedure to remove the fold of skin (the foreskin) that covers the front area of the penis. The circumcision will be done after injecting a local pain medicine at the base of your penis to lessen the amount of pain at the time of the procedure. You may feel pain or discomfort from the needle. In very rare cases, men can have an allergic reaction to the pain medicine.

The procedure will take about 40 minutes. You will be able to rest at the clinic for as long as necessary, normally about 30 minutes after the procedure is adequate. You will be given medicine for pain to take home and directions on how to care for the wound. If you follow these directions, the chances of you having any problems are minimal.

Follow-up visits

You will be required to come back to the clinic about seven days after the circumcision. A doctor will check your penis to make sure there are no problems or bad effects from the circumcision. In case of any problems, you will receive the necessary treatment.

In case you have heavy pain, swelling, bleeding, or any signs of infection that you think are not normal at any time, you should not wait for the seven days to lapse; return to the clinic right away to be checked by the clinicians.

Because it is important for the skin to heal properly after the procedure, you must not have sex for at least six weeks after circumcision.

Risks and discomforts

Like any surgical procedure, there are risks associated with circumcision. The risks include:

- Bleeding
- Swelling
- Pain
- Infection
- In very rare cases, permanent injury, numbness, loss of sensitivity, mutilation, or total loss of the penis There is also the risk of HIV or other infections if you have sex before the wound is fully healed (usually six weeks after the procedure).

You should be aware that circumcision does not fully protect you from HIV or other sexually transmitted infections. Whether circumcised or not, you should protect yourself from HIV by abstaining from sex or being faithful to one partner whom you are sure is HIV negative or by using a condom correctly every time you have sex. Please feel free to ask any questions about the circumcision procedure or about the risks and benefits of circumcision before making your decision to be circumcised in this clinic. After weighing all the factors and you choose to be circumcised, please sign the consent form on the opposite side.
